# Effect of Post-Implantation Heat Treatment Conditions on Photoluminescent Properties of Ion-Synthesized Gallium Oxide Nanocrystals

**DOI:** 10.3390/nano14100870

**Published:** 2024-05-17

**Authors:** Dmitry S. Korolev, Kristina S. Matyunina, Alena A. Nikolskaya, Alexey I. Belov, Alexey N. Mikhaylov, Artem A. Sushkov, Dmitry A. Pavlov, David I. Tetelbaum

**Affiliations:** 1 Research Institute of Physics and Technology, Lobachevsky State University of Nizhny Novgorod, 603022 Nizhny Novgorod, Russia; matyunina.ks@gmail.com (K.S.M.); nikolskaya@nifti.unn.ru (A.A.N.); belov@nifti.unn.ru (A.I.B.); sushkov@phys.unn.ru (A.A.S.); tetelbaum@phys.unn.ru (D.I.T.); 2 Department of Physics, Lobachevsky State University of Nizhny Novgorod, 603022 Nizhny Novgorod, Russia; pavlov@unn.ru; 3 Research and Educational Center “Physics of Solid-State Nanostructures”, Lobachevsky State University of Nizhny Novgorod, 603022 Nizhny Novgorod, Russia; mian@nifti.unn.ru

**Keywords:** gallium oxide, nanocrystals, ion synthesis, ion implantation, thermal annealing, photoluminescence

## Abstract

A novel and promising way for creating nanomaterials based on gallium oxide is the ion synthesis of Ga_2_O_3_ nanocrystals in a SiO_2_/Si dielectric matrix. The properties of nanocrystals are determined by the conditions of ion synthesis—the parameters of ion irradiation and post-implantation heat treatment. In this work, the light-emitting properties of Ga_2_O_3_ nanocrystals were studied depending on the temperature and annealing atmosphere. It was found that annealing at a temperature of 900 °C leads to the appearance of intense luminescence with a maximum at ~480 nm caused by the recombination of donor–acceptor pairs. An increase in luminescence intensity upon annealing in an oxidizing atmosphere is shown. Based on data from photoluminescence excitation spectroscopy and high-resolution transmission electron microscopy, a hypothesis about the possibility of the participation of a quantum-size effect during radiative recombination is proposed. A mechanism for the formation of Ga_2_O_3_ nanocrystals during ion synthesis is suggested, which makes it possible to describe the change in the luminescent properties of the synthesized samples with varying conditions of post-implantation heat treatment.

## 1. Introduction

The rapid development of modern electronic technology means that traditional electronics materials, such as Si or GaAs, no longer meet the increasing demands. Modern semiconductor science faces the challenge of finding materials that can provide qualitatively new properties for use in the next generation of devices. Gallium oxide, an ultra-wide bandgap semiconductor with a bandgap width of more than 4.5 eV, is the most promising candidate as the basic material for prospective electronic devices, since Ga_2_O_3_ has a high breakdown field and is a thermally, chemically, and radiation-resistant material [1,2,3,4,5]. An important advantage of Ga_2_O_3_ is the presence of several polymorphic modifications, which have significantly different physical properties. Usually, five most common phases are distinguished—monoclinic (β-Ga_2_O_3_), defective spinel (γ), rhombohedral (α), cubic (δ), and orthorhombic (ε) [6]. The transition between different phases is mainly realized by varying the annealing conditions; however, finding the optimal heat treatment conditions for a controlled transition between phases is a nontrivial problem. The large value of the bandgap width of Ga_2_O_3_ makes it a promising material for the creation of optoelectronic devices operating in the ultraviolet range of the spectrum [7,8,9,10]. The study of Ga_2_O_3_ optical properties has shown its efficiency for applications in photodetectors [11], light-emitting diodes [12], UV-transparent conductive layers for photolithography [13], and solar energy converters [14], as well as for the creation of scintillators for radiation detection in medical research [15]. However, the difficulty in obtaining monocrystalline wafers and high-quality epitaxial films, as well as the physical limitations inherent in gallium oxide, limit the practical application of this material.

One of the possibilities for solving this problem is the use of Ga_2_O_3_-based nanostructures. At present, the prospect of using such structures for photonic devices [16], solar cells [17], and light-emitting structures [18] has already been demonstrated. The possibility of using Ga_2_O_3_ nanostructures for the creation of low-cost UV-to-visible converters for monitoring UV-emitting events on a large scale—from invisible hydrogen flames to corona dispersions—attracts special attention [18]. However, the chemical methods traditionally used to synthesize such materials are incompatible with conventional CMOS technology. 

Ion-beam synthesis of Ga_2_O_3_ nanoinclusions in dielectric matrices can be a promising approach for obtaining such materials. The non-equilibrium nature of the processes underlying this method, as well as its full compatibility with microelectronics technology, allow us to overcome some disadvantages of Ga_2_O_3_ and provide the possibility of synthesizing structures with specified properties and the ability to control their parameters by varying the conditions of ion irradiation and subsequent heat treatment [19]. Previously, the possibility of ion synthesis of Ga_2_O_3_ nanocrystals (nc-Ga_2_O_3_) was demonstrated, the structure and chemical composition of the samples were studied, and the possibility of obtaining photosensitive and light-emitting structures was demonstrated [20,21,22]. A detailed study of the influence of the ion implantation order on the properties of the synthesized structures was carried out. However, the influence of annealing conditions, which is a key stage in the formation of ion-synthesized nc-Ga_2_O_3_, has not been studied in detail. Meanwhile, heat treatment conditions, such as temperature, duration, and atmosphere, can significantly affect both the structure and chemical composition of nanoinclusions and their photoluminescent properties. Moreover, it has been demonstrated that annealing in an oxygen atmosphere leads to a significant increase in the concentration of Ga-O bonds in the state of stoichiometric oxide Ga_2_O_3_ [22]. In this work, the influence of post-implantation annealing conditions on the photoluminescent properties of gallium oxide nanocrystals ion-synthesized in a SiO_2_/Si dielectric matrix was studied. 

## 2. Materials and Methods

The initial samples were SiO_2_ films with a thickness of 350 nm deposited on *n-Si* (100) substrates via electron-beam evaporation. The ion synthesis process was carried out in two stages. In the first stage, the initial samples were irradiated with gallium (80 keV, 5 × 10^16^ cm^−2^) and oxygen ions (23 keV, 6 × 10^16^ cm^−2^) in different orders. The irradiation regimes were selected based on the coincidence of the distribution profiles of the implanted atoms. A single implantation of Ga^+^ ions only, under the same irradiation conditions, was also used. According to calculations using the SRIM program (www.srim.org), the average projected range of gallium and oxygen ions with the given energies was ~60 nm. The second stage of ion synthesis consists of thermal annealing of ion-irradiated samples. In this work, annealing was carried out in a tube furnace with variation in heat treatment conditions. Some of the samples were annealed sequentially at temperatures of 300, 500, 700, and 900 °C (30 min each) in a nitrogen atmosphere. The other part of the samples was subjected to single (one-step) annealing at temperatures of 700 and 900 °C in a nitrogen atmosphere, as well as at 900 °C in an oxygen atmosphere. The duration of annealing was 30 min.

The photoluminescence (PL) spectra were studied according to the standard technique with synchronous detection. The plasma light source XWS-65 (TRDC, Troitsk, Russia) was used as an excitation source, with a filter separating a ~5 nm wide region from the spectrum, with a maximum at a wavelength of 245 nm. The spectra were recorded at room temperature. In the study of the PL excitation spectra, another grating monochromator was added to the optical scheme, which was used to select the wavelength of the excitation from the same source. The structures of the synthesized samples were studied using high-resolution transmission electron microscopy (HRTEM) on a JEM-2100F microscope (Jeol, Tokio, Japan). The fabrication of samples for cross-section geometry studies was carried out according to the standard technique of Gatan using the 601.07000 TEM Specimen Preparation Kit (Gatan, Pleasanton, CA, USA).

## 3. Results and Discussion

### 3.1. PL Spectra of Irradiated SiO_2_/Si Samples after Annealing in Different Regimes

High-temperature annealing is an important stage in the formation of ion-synthesized nanoinclusions in solid-state matrices. At the same time, heat treatment conditions, such as temperature, duration, and atmosphere, can significantly affect both the structure and chemical composition of nanoinclusions and their photoluminescent properties. The photoluminescent properties of SiO_2_/Si samples implanted only with gallium ions or double implantation of gallium and oxygen ions after sequential annealing at temperatures of 300, 500, 700, and 900 °C (30 min each in a nitrogen atmosphere) were studied earlier in [21]. 

In this work, SiO_2_/Si samples implanted with gallium and oxygen ions in different sequences were investigated, and the effect of post-implantation annealing conditions on the photoluminescent properties of ion-synthesized gallium oxide nanocrystals was studied.

**Figure 1:**
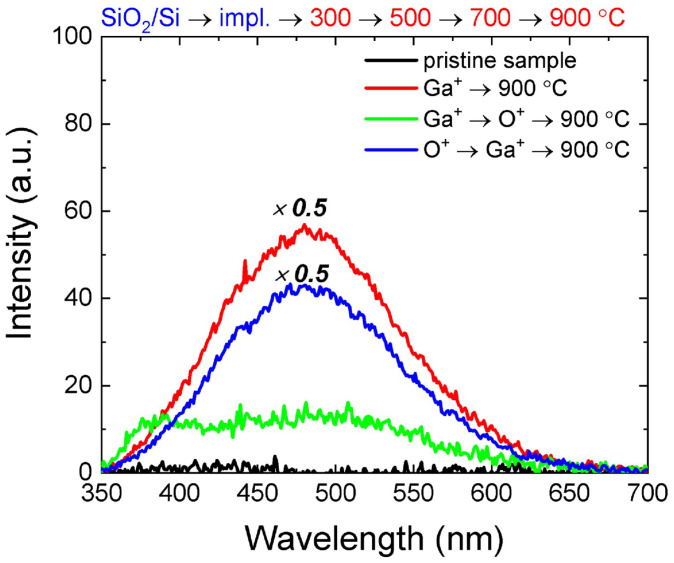
PL spectra of SiO_2_/Si samples irradiated with Ga^+^ and O^+^ ions, subjected to sequential annealing, after final annealing at 900 °C. For comparison, the spectrum of an unirradiated SiO_2_/Si film subjected to annealing under the same conditions is given.

Figure 1 shows the PL spectra of the sequentially annealed samples after final annealing at 900 °C.

The spectra show the appearance of a band in the region of 400–550 nm, and the intensity and shape of the spectral line depend significantly on the implantation order. The highest PL intensity is observed for the sample implanted with gallium ions only. For the sample that was pre-irradiated with oxygen ions before Ga^+^ implantation, the intensity is slightly lower, with the shape of the spectrum repeating that of the sample irradiated with only Ga^+^ ions. The sample in which oxygen was implanted after gallium shows not only a weak PL but also a change in the shape of the spectrum, with a split of the band into two with maxima at ~380 nm and ~480 nm. It was previously shown [21] that the bimodal shape of the spectrum is characteristic of implanted samples subjected to sequential annealing after the annealing stage at 700 °C. To check the influence of heat treatment conditions on the PL spectra of the studied structures, a single annealing was carried out at temperatures of 700 and 900 °C in a nitrogen atmosphere. Also, for comparison, annealing at 900 °C in an oxygen atmosphere was carried out. Single annealing is more preferable from a technological point of view; in addition, such annealing eliminates factors associated with the “thermal history” of the sample, which has passed a series of successive heat treatments. Annealing in an oxygen atmosphere was used to possibly increase the concentration of oxidized gallium, which serves as a basis for the formation of Ga_2_O_3_ nanoinclusions. 

**Figure 2:**
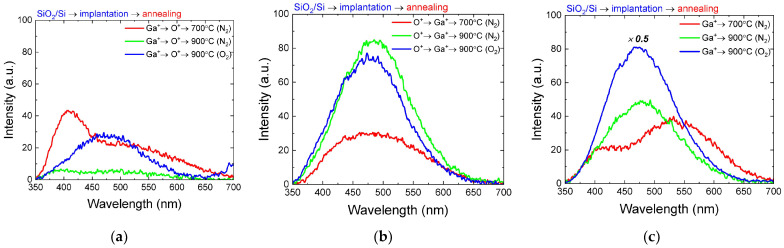
PL spectra of SiO_2_/Si samples irradiated with Ga^+^ and O^+^ ions, subjected to single annealing at temperatures of 700 and 900 °C in different atmospheres: (**a**) samples irradiated in the Ga^+^→O^+^ order; (**b**) samples irradiated in the O^+^→Ga^+^ order; (**c**) samples irradiated only with Ga^+^ ions.

Figure 2 shows the PL spectra of SiO_2_/Si samples irradiated with Ga^+^ and O^+^ ions in different orders, as well as with Ga^+^ ions only, annealed at 700 and 900 °C in a nitrogen atmosphere or at 900 °C in an oxygen atmosphere. For the sample irradiated with oxygen after gallium implantation (Figure 2a), after annealing at 700 °C, a broad bimodal band in the 350–650 nm region is observed on the PL spectrum, which shows two maxima, one of which is located at ~400 nm and the second is located at ~500 nm. Annealing at 900 °C leads to a significant decrease in the PL intensity with preservation of the band shape, and only heat treatment in an oxygen atmosphere at the same temperature leads to the appearance of the PL band with a maximum at ~480 nm.

For the sample irradiated in the O^+^→Ga^+^ order, the shape of the spectrum changes significantly (Figure 2b). After annealing at 700 °C, only one broad peak with a maximum at a wavelength of ~480 nm is observed. Annealing at 900 °C leads to an increase in luminescence intensity by more than two times compared to the sample annealed at 700 °C, and the luminescence intensity and peak position are approximately the same for both inert and oxidation annealing.

The most interesting results were obtained for the sample implanted only with gallium ions without additional irradiation with oxygen ions (Figure 2c). In this case, oxygen from the oxide matrix participates in the formation of Ga-O bonds, which serves as a basis for the formation of Ga_2_O_3_ nanoinclusions, which was demonstrated earlier using X-ray photoelectron spectroscopy [22]. Annealing at 700 °C leads to the appearance of a luminescence band with two maxima at ~410 nm and ~520 nm. The positions of these maxima are red-shifted relative to those in the case of annealing of samples implanted in the Ga^+^→O^+^ order. A band with a single PL maximum at ~480 nm is observed on the sample after annealing at 900 °C in a nitrogen atmosphere. After annealing the sample implanted with Ga^+^ ions only, at 900 °C in an oxygen atmosphere, the intensity of the PL maximum becomes the highest among all the studied samples. At the same time, the shape of the spectrum remains practically the same as that during annealing in a nitrogen atmosphere.

Let us consider what can be associated with the differences in the light-emitting properties of the synthesized samples. For some variants of the samples, in particular, after annealing at 700 °C, the PL spectrum is characterized by the presence of two maxima at wavelengths of ~400 nm and ~510 nm. These luminescent lines were previously observed in single crystals and epitaxial films of gallium oxide [23,24,25]. The nature of the shorter-wavelength line with a maximum at ~400 nm can be due to several factors. One of the possible mechanisms of PL appearance in this spectral region may be radiative recombination due to defects in the SiO_2_ matrix [26]. However, in favor of a small contribution of this mechanism to the resulting spectra, the data of control experiments on the study of irradiated samples without annealing, as well as SiO_2_/Si films without irradiation subjected to sequential annealing, which do not reveal light-emitting properties under the same conditions of the PL study as the other samples, serve as evidence. Another possible mechanism of luminescence in this band could be the recombination of free electrons and self-trapped holes, as verified by experimental and theoretical studies [27]. The long-wavelength part of the PL band with a maximum at ~510 nm can be related to the well-known “green” luminescence in gallium oxide. The data on the nature of this line are rather contradictory, however, experimental and theoretical works show that the emission band located at 2.4 eV is related to V_Ga_ [27,28,29]. 

As the annealing temperature increases up to 900 °C, the spectra take on the form of a broad band with a maximum at a wavelength of ~480 nm. The position of this line is well known in the literature and is related to the blue luminescence of gallium oxide, which is caused by recombination involving donor–acceptor pairs (DAP) [18]. In gallium oxide, the main donor levels are vacancy levels of oxygen in various structural configurations [29], while gallium–oxygen vacancy pair (V_O_, V_Ga_) is believed to be the most likely type of defect that forms an acceptor level [16].

### 3.2. PL Excitation Spectra 

**Figure 3:**
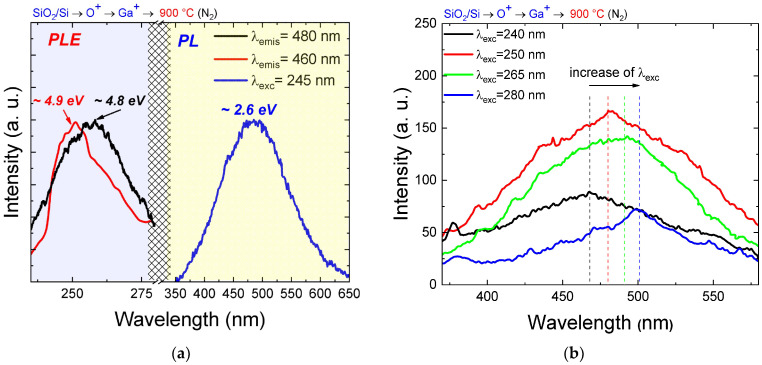
(**a**) PL and PLE spectra of SiO_2_/Si samples irradiated with O^+^→Ga^+^ ions after single annealing at a temperature of 900 °C in a nitrogen atmosphere. PL excitation curves are shown for emission wavelengths of 460 nm (red curve) and 480 nm (black curve). (**b**) PL spectra for different excitation wavelengths.

To obtain additional data on the nature of the observed luminescence, recording PL excitation (PLE) spectra is a promising option. The position of the PLE line can indicate which centers are involved in radiative recombination at the corresponding wavelength. Figure 3 shows the PL spectrum resulting from excitation at a 245 nm wavelength and the PL spectra at different excitation wavelengths. The wide luminescence band ranges from 400 to 550 nm with a maximum at ~480 nm (~2.6 eV), which is typical for the studied samples annealed at 900 °C. For this band, two PL excitation spectra were recorded for two emission wavelengths at 460 and 480 nm (Figure 3a).

The difference between the excitation spectra for different emission wavelengths has been revealed. At a wavelength of 480 nm, the maximum energy of the excitation spectrum is approximately 4.8 eV, while at a wavelength of 460 nm, the maximum energy is around 4.9 eV. These differences can be explained as follows. The PLE spectrum’s maximum position indicates the energy required to photoexcite the carrier to an upper level that participates in radiative recombination. The energy of the maxima corresponds to the typical value of the bandgap of Ga_2_O_3_, providing additional evidence that the observed PL is associated with radiative recombination in gallium oxide. When light with energy greater than the bandgap is applied, it leads to the movement of carriers from the conduction band to the valence band. These carriers are then captured by the donor level in the bandgap, leading to radiative recombination between this level and the acceptor level. The acceptor level involves a gallium vacancy (V_Ga_) and a pair of gallium and oxygen vacancies (V_O_, V_Ga_), as previously mentioned. A comparable mechanism was previously observed for nc-Ga_2_O_3_ obtained through a chemical method [18]. The variation in the position of the maximum excitation energy for different PL wavelengths is most likely due to the size distribution of nc-Ga_2_O_3_. Data on the dependence of the position of the PL maximum on the excitation wavelength shown in Figure 3b demonstrate the presence of a red shift in PL, which further supports the above explanation. In the literature [30,31], a shift in the maximum emission was demonstrated depending on the size of nanocrystals, which was explained by the manifestation of the quantum-size effect. The significant width of the PL band is assumed to be determined by the large scatter in nanoparticle sizes.

### 3.3. Structure of Studied Samples

**Figure 4:**
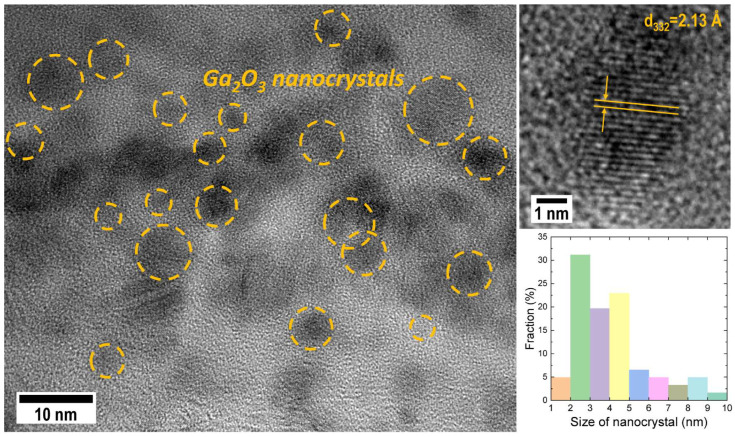
HRTEM image of the SiO_2_/Si sample irradiated with O^+^ and Ga^+^ ions after one-step annealing at 900 °C. The circles in the image show nc-Ga_2_O_3_. The insets provide a magnified view of nc-Ga_2_O_3_ and a histogram illustrating nc-Ga_2_O_3_ size distribution.

The structures of the irradiated samples were studied using high-resolution transmission electron microscopy (HRTEM). Figure 4 shows a cross-section of a SiO_2_/Si sample irradiated with O^+^ and Ga^+^ ions after a single annealing at 900 °C. 

The high-resolution image displays areas exhibiting a periodic structure. Analysis of the images using direct measurement of interplanar distances on TEM images, as well as by analyzing diffraction patterns obtained using the Fourier transform method, reveals that the observed areas are crystalline inclusions with interplanar distances characteristic of gallium oxide. These areas are circled in Figure 4 for clarity. The size spread for the formed nanocrystals is quite large. The histogram in the inset of Figure 4 displays the size distribution of nanoinclusions formed in several areas of the sample. The nanoinclusions have sizes ranging from 1 to 10 nm, with a predominant diameter of 2–5 nm. This confirms the observed dependence of the luminescence excitation length on the position of the PL emission line, which is likely due to the involvement of Ga_2_O_3_ nanoparticles of varying sizes in radiative recombination. 

## 4. Discussion

**Figure 5:**
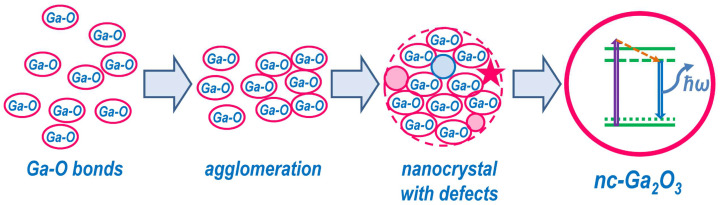
Schematic representation of the proposed formation mechanism of light-emitting nc-Ga_2_O_3_.

The obtained data indicate that changes in the light-emitting properties of gallium oxide nanoinclusions are significantly dependent on the annealing conditions. This can be explained by the fact that PL lines in the region of 400–550 nm do not appear for all synthesis options during sequential annealing at temperatures of 300 and 500 °C, or the intensity of these lines was low, as previously shown [21]. However, the PL intensity increased significantly after the final annealing at a temperature of 900 °C, except for the case of irradiation with ions in the order Ga^+^→O^+^. This sample is characterized by the presence of two maxima in the PL spectrum, along with low intensity. Let us now consider a possible explanation of the observed difference. During the process of ion synthesis, Ga-O bonds form, which act as the “seeds” for the formation of Ga_2_O_3_ nanoinclusions. This process occurs even without annealing. Heat treatment results in the formation of gallium oxide precipitates. As the annealing temperature increases, these precipitates grow and form “non-phase” inclusions, which may exhibit light-emitting properties. An increase in temperature results in the creation of Ga_2_O_3_ nanoinclusions that may contain numerous defects. Subsequently, annealing at approximately 900 °C leads to the formation of Ga_2_O_3_ nanocrystals with a relatively low concentration of defects. Figure 5 illustrates this mechanism. It is worth noting that the proposed mechanism for the formation of nanocrystals is only a hypothesis that requires additional experimental verification.

By considering the proposed model for the formation of nc-Ga_2_O_3_, it is possible to explain the observed changes in the PL spectra during annealing. In the case of annealing at 700 °C, “non-phase” inclusions of gallium oxide predominate in the synthesized samples, depending on the order of irradiation, and the process of nanoinclusion synthesis is at the initial stage. In this case, the PL spectra show peaks at approximately 400 and 510 nm, which are believed to be caused by recombination involving elementary defects that is more likely to occur in highly defective structures. However, the PL intensity remains low, indicating a small number of formed nanoinclusions and a high concentration of defects, which act as centers of nonradiative recombination. Raising the annealing temperature to 900 °C completes the formation of nanocrystalline Ga_2_O_3_ inclusions, resulting in the observation of a single PL line at approximately 480 nm, and its position is practically independent of the synthesis conditions. An exception is the PL spectra for the samples irradiated with oxygen after irradiation with gallium. This is due to the fact that, for these samples, a lower gallium content is observed, especially in the oxidized state compared to the other implantation options used, which may be due to gallium outdiffusion [22]. Therefore, for these samples, the completion of the formation process of nc-Ga_2_O_3_ occurs only after annealing in an oxygen atmosphere, which contributes to an increase in the proportion of oxidized gallium and, apparently, to an increase in the number of light-emitting particles. 

The PL spectra of the studied samples commonly exhibit a broad luminescence bandwidth. This bandwidth may be attributed to the size dispersion of the synthesized nc-Ga_2_O_3_, as demonstrated through the HRTEM method used to study the sample structure. Supporting this mechanism is the data from PL excitation spectroscopy, which indicates that the excitation energy is dependent on the emission line position. This effect may be attributed to the quantum-size effect, which is possible in structures with synthesized nanoclusters. However, to verify this hypothesis, detailed theoretical calculations for such a system are necessary, which will be the subject of a separate study.

## 5. Conclusions

The influence of post-implantation heat treatment conditions on the luminescent properties of ion-synthesized Ga_2_O_3_ nanocrystals in a SiO_2_/Si matrix was studied. It was demonstrated that changes in heat treatment conditions (temperature and annealing atmosphere) significantly affected the photoluminescence spectra. Thus, for annealing at a temperature of 700 °C, for some of the implantation conditions used (the order of ion irradiation), two luminescence peaks are observed, associated with the recombination at defect-related states. Increasing the annealing temperature to 900 °C leads to a transformation of the spectrum with the appearance of one maximum at a wavelength of ~480 nm associated with the recombination of donor–acceptor pairs, where a gallium vacancy (V_Ga_) and a pair of gallium and oxygen vacancies (V_Ga_, V_O_) act as a donor and acceptor, respectively. Changing the annealing atmosphere from an inert to an oxidizing one has a positive effect on the luminescence intensity, which may be due to an increase in the concentration of gallium and oxygen bonds in the state of stoichiometric oxide Ga_2_O_3_, which contributes to an increase in the number of light-emitting nanoparticles. 

The use of photoluminescence excitation spectroscopy techniques allowed us to suggest a hypothesis about its mechanism. A dependence of the excitation energy on the radiation wavelength was discovered, which may indicate a quantum-size effect. Additional confirmation in favor of this assumption is provided by transmission electron microscopy data, confirming the formation of gallium oxide nanocrystals in the studied structures with a large spread in size and an average diameter of ~2–5 nm.

Based on the obtained experimental data, a possible mechanism for the formation of gallium oxide nanocrystals in the process of ion synthesis is proposed, which includes the stages of Ga-O bonds formation, their agglomeration, the formation of defect inclusions and, finally, Ga_2_O_3_ nanocrystals, the transition between which occurs with increasing annealing temperature.

Thus, the performed experiments demonstrate the promise of the ion synthesis technique for obtaining light-emitting nanostructures based on gallium oxide. Ion implantation provides a richer arsenal of ways to control the luminescent properties of nanostructures compared to other methods but requires greater knowledge about the kinetics of defects and their behavior during the annealing process. The obtained results can be used to develop modern optical devices operating in the UV and visible spectral regions. The developed approach to the synthesis of gallium oxide nanocrystals opens up prospects for the use of such structures not only as light-emitting ones but also in the creation of highly efficient UVC photodetectors [32]. A significant advantage of this technique is its full compatibility with modern CMOS technology.

## Data Availability

The data presented in this study are available on request from the corresponding authors.
